# Evaluation of lenticular antioxidant and redox system components in the lenses of acetyl-L-carnitine treatment in BSO-induced glutathione deprivation

**Published:** 2009-07-31

**Authors:** R. Elanchezhian, M. Sakthivel, M. Isai, P. Geraldine, P.A. Thomas

**Affiliations:** 1 Department of Animal Science, School of Life Sciences, Bharathidasan University, Tiruchirappalli, Tamil Nadu, India; 2 Institute of Ophthalmology, Joseph Eye Hospital, Tiruchirappalli, Tamil Nadu, India

## Abstract

**Purpose:**

To investigate whether acetyl-L-carnitine (ALCAR) retards L-buthionine-(S,R)-sulfoximine (BSO)-induced cataractogenesis in Wistar rat pups.

**Methods:**

On postpartum day 3, group I pups received intraperitoneal (ip) saline and group II and group III pups received i.p. injections of BSO once daily for three consecutive days. In addition, group III pups received ip ALCAR once daily from postpartum days 3–15. Both eyes of each pup were examined up from postpartum day 16 to day 30. After sacrifice, extricated pup lenses were analyzed for antioxidant and redox system components.

**Results:**

There was dense lenticular opacification in all group II pups, minimal opacification in 40% of group III pups, and no opacification in 60% of group III pups and in all of group I pups. Group II lenses exhibited significantly lower values of antioxidant and redox system components and higher malondialdehyde concentrations than in group I or group III lenses.

**Conclusions:**

ALCAR prevents cataractogenesis in the BSO-induced cataract model, possibly by inhibiting depleting antioxidant enzyme and redox system components and inhibiting lipid peroxidation.

## Introduction

Oxidative stress is a common initiator of many age-related conditions and is probably the most important mechanism in age-related cataractogenesis. Aging of the lens is associated with progressive changes in the physical and chemical properties of its structural proteins, the crystallins. Oxidative stress-induced changes include crystallin cross-linking, aggregation, loss of solubility, conformational alterations, fragmentation, and enzyme inactivation. Highly reactive species such as hydrogen peroxide (H_2_O_2_), singlet oxygen, superoxide radicals, and hydroxyl radicals can be generated in the eye through photochemical pathways [1] or Fenton-type reactions [2]. The lens possesses several protective mechanisms to prevent or limit oxidative damage. Normal young lenses maintain optimal activity of antioxidant enzymes and high concentrations of ascorbate and glutathione and hence minimize the alterations wrought by excessive oxidation. If this balance of pro- and antioxidants is disturbed, aging occurs. Under the pathological condition, the oxidation of lenticular proteins may lead to senile cataract [3].

Glutathione is a major constituent of mammalian lenses and is mainly concentrated in the epithelium. Glutathione decreases during the formation of most cataracts [4]. Reduced glutathione (GSH) is present in a high concentration in the lens [5,6]. GSH serves as an intracellularly-produced antioxidant, which resists oxidative damage to cellular organelles by recycling other antioxidants, scavenging free radicals, and using H_2_O_2_  and hydroperoxides where it undergoes oxidation by glutathione peroxidase [7,8]. It also promotes the antioxidant properties of vitamin C and vitamin E by maintaining these nutrients in a reduced state [9]. The second line of defense for the health of the lens is its content of intrinsic repair enzymes that constantly dethiolate protein-thiol mixed disulfides (protein thiolation) or protein–protein disulfides, which have been induced by oxidative stress. This process allows lenticular proteins to maintain their free thiols again, thus restoring lenticular proteins as well as the function and activities of enzymes [10]. The role of GSH as an endogenous lenticular antioxidant results in the reduction of lenticular hydrogen peroxide [11] and dehydroascorbate [12].

L-buthionine-(S,R)-sulfoximine (BSO), an inhibitor of GSH biosynthesis, can induce age-dependent cataracts in pre-weaning mice [13] and in early postnatal rats [14] and is thus a potential model for obtaining new information about the role of GSH in maintaining transparency of the lens. In the presence of reduced levels of GSH, newborn rats suffer extensive damage to the cytosolic proteins and membrane lipids, leading to clouding of the lens [15-17]. BSO-induced cataracts have been prevented or reduced in frequency in vivo by esters of GSH [12,14] and by lipoic acid [18] and ascorbate [12].

Acetyl-L-carnitine (ALCAR), a quaternary amine, is a naturally-occurring, short-chain derivative of L-carnitine, which is synthesized endogenously in the human brain, liver, and kidneys by the acetyl carnitine transferase enzyme or obtained from dietary sources [19]. ALCAR facilitates the uptake of acetyl-CoA into the mitochondria during fatty acid oxidation, enhances acetylcholine production, and stimulates synthesis of protein and membrane phospholipids. It also counteracts oxidative stress by inhibiting increases in lipid hydroperoxidation [20]. ALCAR has been reported to prevent selenite-induced cataractogenesis in a Wistar rat model both in vitro and in vivo by maintaining lenticular antioxidant and redox system components [21,22] and lenticular calpain activity [23] at near normal levels. In this study, an attempt has been made to determine the putative anticataractogenic effect of ALCAR by preventing the depletion of glutathione in the BSO-induced cataract model. Certain key biochemical parameters of antioxidant and redox system components and of lipid peroxidation have also been evaluated.

## Methods

### Experimental animal

Two-day-old rat pups (Wistar strain) were used in this study. The pups were housed with parents in large spacious cages, and the parents were given food and water ad libitum. The animal room was well ventilated, and a regular 12 h light and 12 h dark cycle was maintained throughout the experimental period. These animals were used in accordance with institutional guidelines and with the Association for Research in Vision and Ophthalmology Statement for the Use of Animals in Research. The rat pups were divided into three groups, each group comprising pups from the same litter: Group I, which received only saline (control); Group II, which received BSO (cataract-untreated); Group III, which received BSO and ALCAR (cataract-treated).

Each rat pup in groups II and III received an intraperitoneal (ip) injection of BSO once daily for three consecutive days starting from postpartum day 2. In addition, pups in group III received ip injections of ALCAR (200 mg/kg bodyweight), which was administered half an hour before the BSO injection once a day until the pups opened their eyes.

### Morphological examination

When the rat pups first opened their eyes, a slit-lamp biomicroscopic examination was performed on each eye to detect opacification. Prior to performing the examination, mydriasis was achieved by a topical ophthalmic solution, which was instilled every 30 min for 2 h with the animals being kept in a dark room. After 2 h, the eyes were viewed by a slit-lamp biomicroscope (Carl Zeiss, Jena, Germany) at 12X magnification. At the end of the experimental period (postpartum day 30), each eye was photographed, and the degree of opacification was graded as follows: 0=normal transparent lens; +=initial sign of nuclear opacity involving tiny scatters; ++=partial nuclear opacity; and +++=dense nuclear opacity.

### Biochemical evaluation of redox system components and antioxidant enzymes

Rat pups in all three groups were anesthetized and then sacrificed by cervical dislocation on postpartum day 30. The lenses were then excised. Both lenses of each individual rat were processed together to constitute a single value. The lenses were homogenized in 50 mM phosphate buffer (pH 7.2; 1 ml/ 100 mg tissue) and centrifuged at 14,006x g for 15 min at 4 °C. The supernatant obtained was used for the analysis of enzymatic and non-enzymatic parameters. To calculate the specific enzyme activity, protein in each sample was estimated by the method of Bradford [24].

### Reduced glutathione

The GSH content was estimated by the method of Moron et al. [25]. The lens homogenate was centrifuged at 2,432x g for 15 min at 4 °C. To the resulting supernatant, 0.5 ml of 10% trichloroacetic acid was added, and the mix was recentrifuged. The resulting protein-free supernatant was allowed to react with 4 ml of 0.3 M Na_2_HPO_4_ (pH 8.0) and 0.5 ml of 0.04% (wt/vol) 5,5-dithiobis-2-nitrobenzoic acid. The absorbance of the resulting yellow color was read spectrophotometrically at 412 nm. A parallel standard was also maintained. The results were expressed in μmoles/g wet weight.

### Glutathione reductase

This enzyme, which utilizes nicotinamide adenine dinucleotide phosphate (NADPH) to convert oxidized glutathione to the reduced form, was assayed by the method of Stall et al. [26]. The change in absorbance was read at 340 nm for 2 min at intervals of 30 s in an ultraviolet (UV) spectrophotometer (Analytik Jena AG, Jena, Germany). The activity of glutathione reductase (GR) was expressed as nmoles of NADPH oxidized/min/mg protein.

### Glutathione S-transferase

The activity of glutathione S-transferase (GST) was determined by the method of Habig and Jacoby [27]. The conjugation of GSH with 1-chloro-2,4-dinitrobenzene (CDNB), a hydrophilic substrate, was observed spectrophotometrically at 340 nm to measure the activity of GST. One unit of GST was defined as the amount of enzyme required to conjugate 1 μmol of CDNB with GSH per min.

### Glutathione peroxidase

The activity of glutathione peroxidase (GPx) was determined essentially as described by Rotruck et al. [28]. The principle of this method is that the rate of glutathione oxidation by H_2_O_2_ as catalyzed by the GPx present in the supernatant is determined. The color that develops is read against a reagent blank at 412 nm on a spectrophotometer. In the test, the enzyme activity was expressed as units/mg protein (one unit was the amount of enzyme that converted 1 µmole of GSH to the oxidized form of glutathione [GSSH] in the presence of H_2_O_2_ per min).

### Catalase

Catalase (CAT) activity was determined by the method of Sinha [29]. In this test, dichromatic acetic acid is reduced to chromic acetate when heated in the presence of H_2_O_2_ with the formation of perchloric acid as an unstable intermediate. In the test, the green color developed was read at 590 nm against a blank on a spectrophotometer. The activity of catalase was expressed as units/mg protein (one unit was the amount of enzyme that used 1 mmole of H_2_O_2_ per min).

### Superoxide dismutase

Superoxide dismutase (SOD) activity was determined by the method of Marklund and Marklund [30]. In this test, the degree of inhibition of pyrogallol auto-oxidation by the supernatant of the lens homogenate was measured. The change in absorbance was read at 470 nm against the blank each min for 3 min on a spectrophotometer. The enzyme activity was expressed as units per mg protein.

### Determination of lipid peroxidation

The extent of lipid peroxidation was determined by the method of Ohkawa et al. [31]. The principle of this method is that malondialdehyde (MDA), an end-product of lipid peroxidation, reacts with thiobarbituric acid (TBA) to form a pink chromogen. For this assay, 0.2 ml of 8.1% sodium dodecyl sulfate, 1.5 ml of 20% acetic acid (pH 3.5), and 1.5 ml of 0.81% thiobarbituric acid aqueous solution were added in succession in a reaction tube. To this reaction mixture, 0.2 ml of the lens homogenate was added, and the mixture was then heated in boiling water for 60 min. After cooling to room temperature, 5 ml of the butanol:pyridine (15:1 v/v) solution were added. The mixture was then centrifuged at 2,432x g for 15 min and the upper organic layer was separated. The intensity of the resulting pink color was then read at 532 nm, and the result was expressed as nmoles of MDA formed per gram wet weight.

### Statistical analysis

The mean value of each parameter in each individual group of rats was calculated from at least five individual values and was expressed as mean±SD. Statistical analysis was done by using the Student’s *t*-test and χ^2^ test where appropriate, and p values less than 0.05 were considered statistically significant.

## Results

### Morphological examination

**Figure 1:**
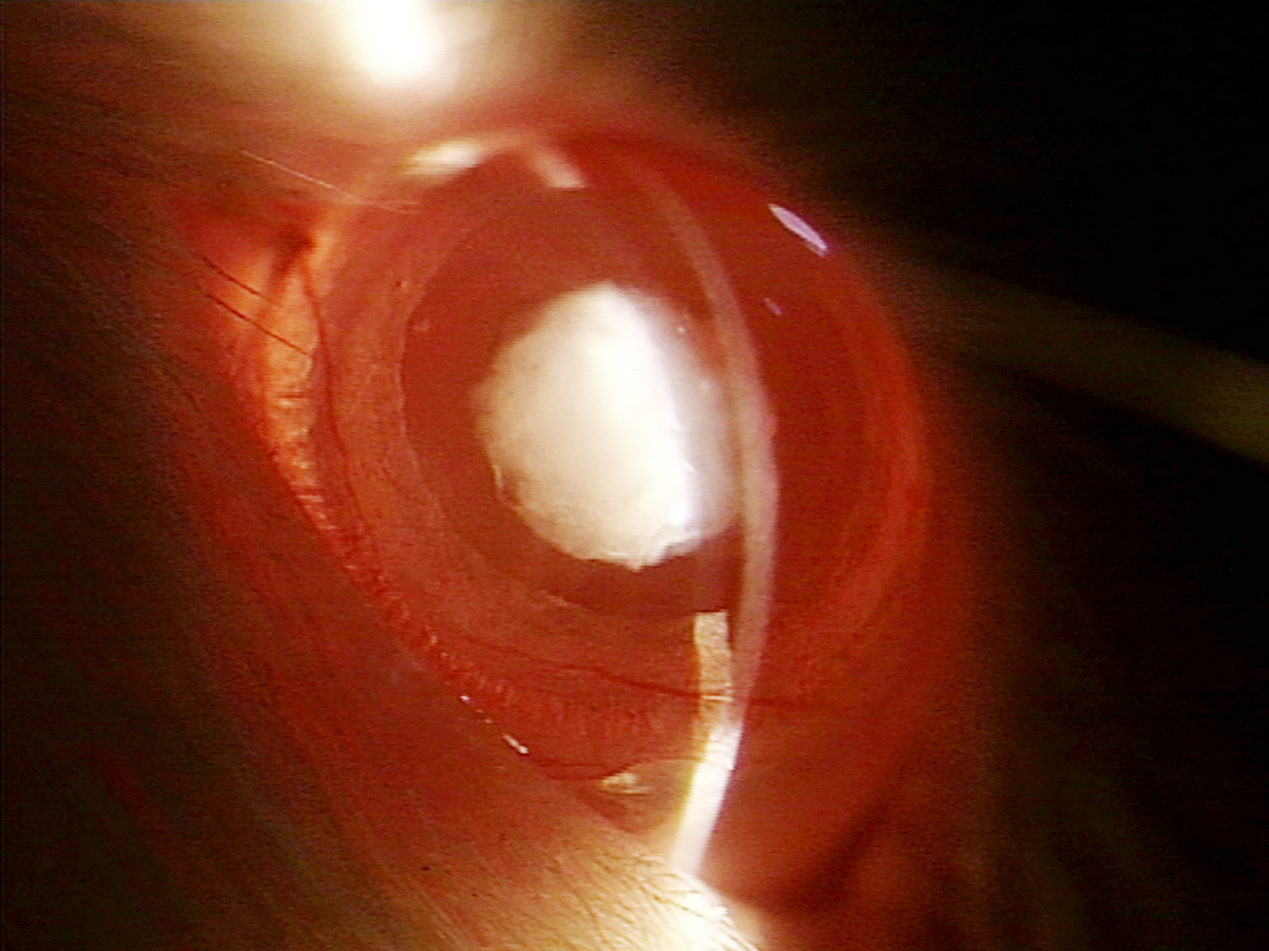
Slit-lamp appearance of the eye of a 30-day-old Wistar rat pup in group II. The eye exhibited dense opacification of the lens (Grade +++ opacification).

**Figure 2:**
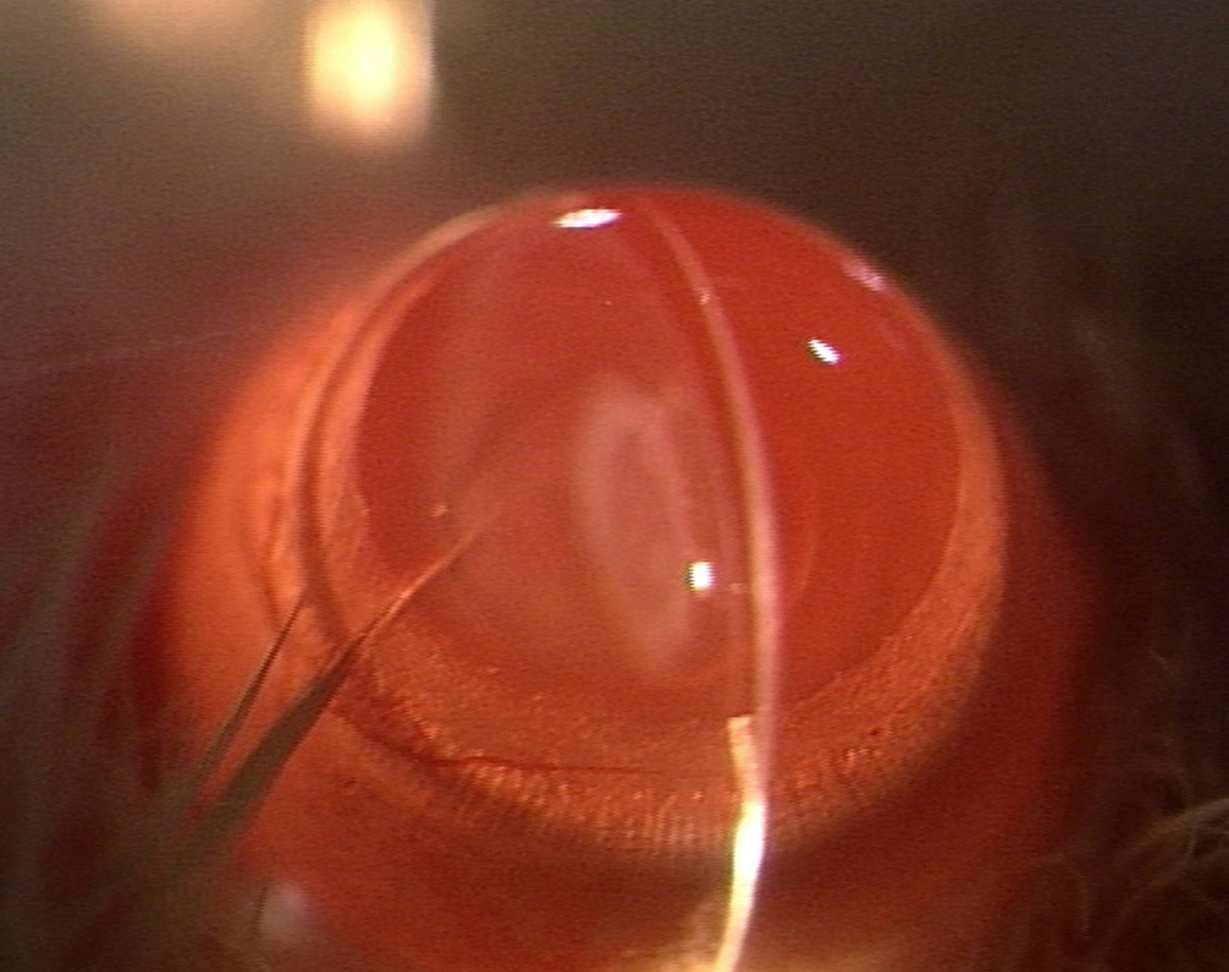
Slit-lamp appearance of the eye of 30-day-old Wistar rat pup in group III. This eye exhibited only slight opacification of the lens (Grade + opacification).

**Figure 3:**
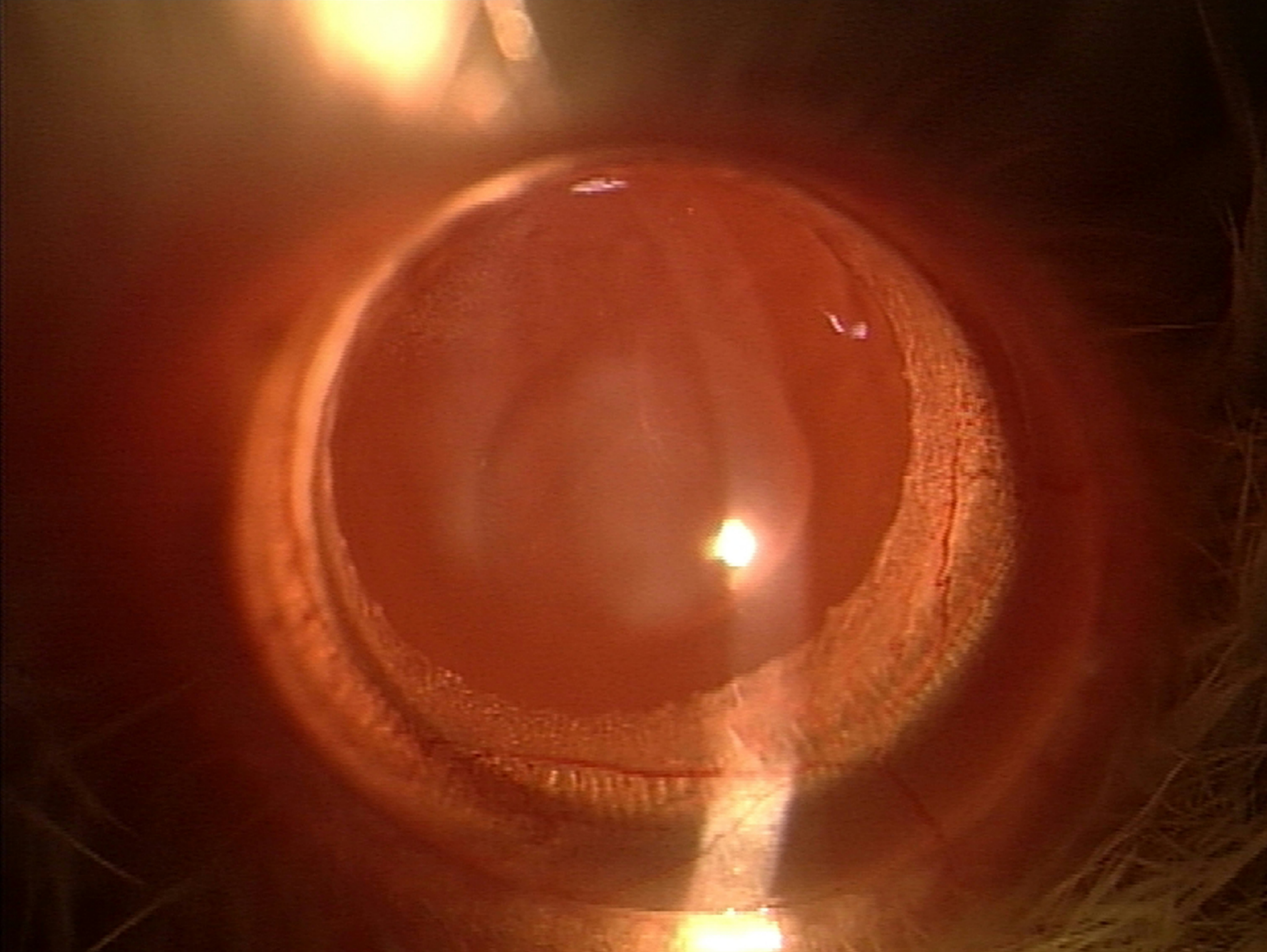
Slit-lamp appearance of the eye of 30-day-old Wistar rat pup in group I. This eye exhibited no opacification of the lens (Grade 0 opacification).

Slit-lamp examination revealed that all 15 rat pups in group II (Figure 1 and Table 1) exhibited dense opacification of the lens (grade +++). In contrast, only 6 of 15 (40%) rat pups in group III (Figure 2 and Table 1) exhibited lenticular opacification (grade +) with the lenses of the other nine pups appearing normal (grade 0). All 15 rat pups in group I exhibited maximum transparency (grade 0) of the lens (Figure 3 and Table 1). The difference between the value in group II and group III rats was statistically significant (χ^2^ [degrees of freedom=1]=12.8; p<0.01).

**Table 1:** Morphological examination of lenses of rat pups.

**Experimental Groups**	**Number of pups**	**Number of pups with different degrees of lenticular opacification**				**Number of pups in which lenticular opacification occurred**
		**0**	**+**	**++**	**+++**	
**Group I** (normal)	15	15	-	-	-	0
**Group II** (cataract-untreated)	15	-	-	-	15	all 15
**Group III** (cataract-treated)	15	9	6	-	-	6 of 15 (40%)

Group I rat pups received only saline. Group II rat pups received only L-buthionine-(S,R)-sulfoximine (BSO). Group III rat pups received BSO and acetyl-L-carnitine. The degree of opacification was graded as follows: 0-normal transparent lens; +-initial sign of nuclear; opacity involving tiny scatters; ++-partial nuclear opacity; and +++- mature nuclear opacity.

### Biochemical evaluation of lenticular antioxidant and redox system components

#### Redox system components

The mean activities of GR, GST, and GPx and the mean level of GSH in lenses of BSO-injected rats (group II) were significantly lower than those in lenses of normal rats (group I) (p<0.05; Table 2). The mean activities of GR, GST, and GPx and the mean level of GSH were significantly higher in lenses of group III rats than those in lenses of group II rats (p<0.05). However, no significant differences were observed in the mean activities of GR, GST, and GPx and the mean level of GSH between lenses of normal rats (group I) and the lenses of ALCAR-treated rat lenses (group III; Table 2).

**Table 2:** Quantitative analysis of redox system components in the lenses of rat pups.

**Component analyzed (unit of activity)**	**Group I (normal)**	**Group II (cataract-untreated)**	**Group III (cataract-treated)**
**Reduced glutathione** (μmoles/gram tissue)	8.14±0.10	4.6±0.73*	7.36±0.40*
**Glutathione reductase** (nmoles of NADPH oxidized/min/mg protein)	0.183±0.07	0.115±0.06*	0.168±0.07*
**Glutathione-S-transferase** (μmoles of CDNB conjugated with GSH/min)	5.50±0.32	2.89±0.38*	4.5±0.44*
**Glutathione peroxidase** (μmoles glutathione oxidized/mg protein/min)	43.41±4.9	25.41±1.59*	34.59±1.67*

Each value represents the mean (±SD) of five observations. Statistical analysis was performed by the Student *t*-test. The asterisk indicates statistical significance (p≤0.05) in at least one cut-off level between the group II and group I lenses and between group III and group I lenses. Abbreviation: GSH=Reduced glutathione; NADPH=Nicotinamide adenine dinucleotide phosphate; CDNB=1-chloro-2,4-dinitrobenzene.

#### Antioxidant enzymes

The mean activities of CAT and SOD in lenses of BSO-injected rats (group II) were significantly lower than the values in lenses of normal rats (group I) that had received saline alone (p<0.05; Table 3). Treatment with ALCAR appeared to exert a beneficial effect since the activities of CAT and SOD were significantly (p<0.05) higher in lenses of group III rats than group II rats (Table 3).

**Table 3:** Quantitative analysis of antioxidant enzyme parameters in the lenses of rat pups.

**Enzyme analyzed (unit of activity)**	**Group I (normal)**	**Group 2 (cataract-untreated)**	**Group 3 (cataract-treated)**
**1. Catalase** (μmoles hydrogen peroxide consumed/mg protein/min)	7.45±0.33	4.5±0.47*	6.54±0.31*
**2. Superoxide dismutase** (units/mg protein)	2.35±0.25	0.88±0.08*	1.66±0.11*

Each value represents the mean (±SD) of five observations. Statistical analysis was performed by the Student *t*-test. The asterisk indicates statistical significance (p≤0.05) in at least one cut-off level between the group II and group I lenses and between group III and group I lenses.

#### Determination of lipid peroxidation

**Figure 4:**
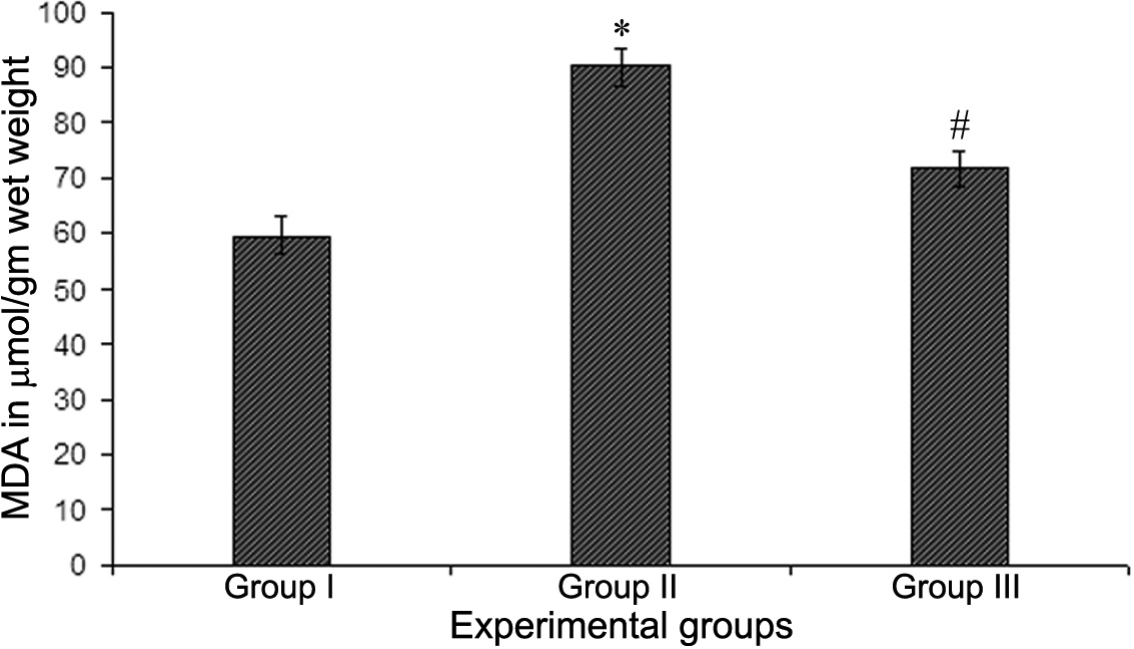
Concentration of malondialdehyde in lenses of 30-day-old Wistar rat pups. Values are expressed as mean±SD (n=5). An asterisk indicates that a significant difference was found between group I and group II values (p≤0.05). The sharp (hash mark) indicates that a significant difference was found between group II and group III values (p≤0.05).

The mean MDA level was found to be significantly higher in lenses of BSO-injected rats (group II) than in normal rats (group I; p<0.05; Figure 4). However, the mean MDA level in group III rat lenses (treated with ALCAR) was significantly lower than in group II rat lenses (p<0.05), presumably due to limitation of lipid peroxidation.

## Discussion

Cataract formation is associated with oxidative insults such as loss of lenticular glutathione, excessive H_2_O_2_, accumulation of lipid peroxides, and lack of oxygen-detoxifying enzymes [32-34]. Humans exposed to hyperbaric oxygen have been found to develop cataract [35]. The role of such oxidative insults in cataractogenesis led us to investigate the role of ALCAR, a known antioxidant, in the prevention of lenticular opacification in newborn rats exposed to BSO. Gross morphological examination appeared to suggest that ALCAR is able to significantly retard BSO-induced cataractogenesis since 100% of rats receiving BSO alone developed dense lenticular opacification while 60% of rats receiving BSO and ALCAR did not develop any lenticular opacification (Figure 1, Figure 3; Table 1).

A high concentration of GSH, a major intracellular antioxidant, has been found to protect the lens from oxidative damage due to toxic chemicals [36]. Thus, depletion of GSH seriously affects GSH-dependent enzymes such as GPx, GR, and GST as well as leukotriene C4 synthetase and the glutaredoxin system, which renders the cells to be susceptible to a toxic challenge [37]. GR maintains the intracellular level of GSH by preserving the integrity of cell membranes and by stabilizing the sulfhydryl groups of proteins. Administration of carnitine and lipoic acid to aged rats has been found to increase the activity of GR by increasing the levels of GSH and the reducing equivalent of NADPH [38,39].

Depletion of GSH appears to be the prime cause of BSO-induced cataract [13]. In the present study, the levels of redox system components (GSH, GR, and GST) were found to be significantly lower in lenses of BSO-administered rats than in normal rat lenses (Table 3). These lowered activities were possibly due to the depletion of the lenticular GSH pool that occurred as a consequence of exposure to BSO. Similar observations have already been reported [40,41]. In the lenses of group III rats (exposed to BSO and treated with ALCAR), the mean level of GSH and the mean activities of GR and GST were found to be significantly higher than the values in the lenses of rats that were administered BSO alone (Table 3). Similar observations have been reported in the selenite-induced cataract model [22]. In fact, the GSH/GPx system has been known to function as an antioxidant system in the mitochondria and cytoplasm of lens epithelial cells. The depletion of lenticular GSH in animals receiving BSO alone and the increased level of GSH following administration of ALCAR may be due to improved energy metabolism, inhibition of electron leakage from mitochondrial electron transport systems [42], and enhanced repair of oxidized membrane/lipid bilayers [43,44], thereby maintaining lenticular GSH levels.

CAT, SOD, and GPx are important components of the innate antioxidant enzymatic defenses of the lens. CAT is able to detoxify significant amounts of H_2_O_2_ [45]. SOD catalyzes the removal of superoxide radicals (O_2_^-^), which would otherwise damage the membrane and other biological structures [46]. The enzyme, GPx, first demonstrated in the lens by Pirie [47], has been reported to maintain the integrity of the phospholipid bilayer of membranes by inhibiting lipid peroxidation. Thus, CAT and GPx catalyze the transformation of H_2_O_2_ within the cell to harmless by-products, thereby curtailing the quantity of cellular destruction inflicted by products of lipid peroxidation. A reduction in the activities of these enzymes in tissues has been associated with the accumulation of highly reactive free radicals, leading to loss of the integrity and function of cell membranes [45,48,49]. In the present in vivo study, the mean activities of CAT, SOD, and GPx were found to be significantly lower in the lenses of rats exposed to BSO alone than those in normal rat lenses (Table 2). Such a reduction in the activities of these enzymes in BSO-induced cataractogenesis has been reported in vitro [50] and in vivo [18,51]. However, in the lenses of rats that had been exposed to BSO and treated with ALCAR, the activities of these enzymes were maintained at near normal levels.

The excessive generation of free radicals leads to peroxidative changes that ultimately result in enhanced lipid peroxidation [52], which causes changes in biochemical processes and structural integrity and leads to cellular damage [53]. In the present investigation, the mean level of lenticular MDA was found to be significantly higher in rats that had been administered BSO alone than in normal rats. However, the mean levels of MDA were significantly lower in lenses of group III rats (BSO-administered and ALCAR-treated) than in group II rat lenses (Figure 4). Thus, lenses of rats given BSO alone showed a significant depletion of GSH and increased membrane damage as indicated by the increased levels of MDA (Figure 4). However, ALCAR appeared to prevent the occurrence of such changes. Similar protective effects of ALCAR have been previously reported in selenite-challenged rat lenses [22].

We have previously reported that ALCAR appears to prevent selenite-induced cataractogenesis [22]. The results of the present study add support to our hypothesis that ALCAR can also prevent cataractogenesis that is mediated by glutathione deprivation and induced by BSO. These preventive effects of ALCAR are suggested by its ability to maintain lenticular antioxidant and redox system components at near normal levels and to prevent excessive lipid peroxidation. The relevance of these results in the context of human senile cataractogenesis requires further study.
